# Predictors of outcome in early onset schizophrenia: a 10-year follow-up study

**DOI:** 10.1186/s12888-020-2484-x

**Published:** 2020-02-14

**Authors:** Lingzi Xu, Yanqing Guo, Qingjiu Cao, Xue Li, Ting Mei, Zenghui Ma, Xinzhou Tang, Zhaozheng Ji, Liu Yang, Jing Liu

**Affiliations:** Peking University Sixth Hospital, Peking University Institute of Mental Health, NHC Key Laboratory of Mental Health (Peking University), National Clinical Research Center for Mental Disorders (Peking University Sixth Hospital), 51 Huayuanbei Road, Haidian District, Beijing, 100191 China

**Keywords:** Early onset schizophrenia, Follow-up, Patient function

## Abstract

**Background:**

Younger age at onset is generally thought to be a predictor of poor outcome in Early Onset Schizophrenia (EOS), but there is a paucity of epidemiological data supporting this belief. This study aims to describe long-term outcomes and predictors of patient functioning in EOS, with a focus on the effect of age at onset.

**Methods:**

We consecutively enrolled 118 EOS patients who were hospitalized in 2006. Mean age at baseline was 13.3 ± 2.3 years. Sixty-five subjects were successfully interviewed. Mean length of follow up was 10.4 ± 0.3 years. Baseline data were collected from inpatient medical records, while follow up was conducted primarily through telephone interviews of patient relatives. WHODAS 2.0 was used to measure global functioning at follow up. Outcomes included education, employment, marriage status, physical health, subsequent diagnoses and treatment, and patient functioning. Univariate and multivariate regression models were used to assess predictors of outcome, while propensity scores were used to adjust for confounding in analyzing the effect of age at onset on functional outcome.

**Results:**

Of the 65 subjects where follow-up data were available, 3 were deceased at follow up. Five (8%) discontinued treatment. Diagnostic stability was 76%. Nearly a quarter (24%) were using clozapine at follow up. In male and female patients, 61 and 55% respectively were overweight, while 29 and 32% respectively were obese. Sixteen (26%) were economically self-sufficient, while 34 (55%) were unemployed. Thirteen (21%) patients had ever been married. The median WHODAS score was 15 (IQR 2 to 35), roughly corresponding to the 78th percentile on population norms. Extroverted personality (*p* = 0.01), suspicious personality (*p* = 0.02), and high level of education (*p* = 0.001) predicted better functioning. Age of onset was not associated with function in either the univariate model (*p* = 0.24), full model (*p* = 0.17) or the final risk factor model (*p* = 0.11), nor after using propensity scores to further adjust for confounders.

**Conclusion:**

The long-term functional outcome of EOS is more optimistic than generally believed. Age at disease onset does not predict long-term functional outcome in EOS populations.

## Background

Schizophrenia is a heterogeneous disease that typically manifests in adolescence or early adulthood. Early onset schizophrenia (EOS) is defined as schizophrenia with onset before 18 years of age, while those with onset before age 13 are sometimes categorized as Very Early Onset Schizophrenia (VEOS) [1]. Traditionally, early age at onset, especially onset before age 13, is believed to be a predictor of poor prognosis [1]. However, there is a paucity of follow up studies in EOS populations. Many existing studies reported only descriptive outcomes [2–4], limiting the clinician’s ability to make evidence-based treatment decisions. Though some studies explored predictive factors of long term outcome [5, 6], results were not conclusive due to small sample sizes, relatively heterogeneous study populations that included schizophrenia spectrum disorders, and limitations in statistical methods. Furthermore, only one outcome study [4] was conducted outside of high-income Western countries.

The present study aims to describe the 10-year outcomes of EOS patients after discharge from hospitalization, and to explore risk factors related to patient functioning at follow up. Our goal is to fill a gap in the knowledge about long term EOS outcomes in Asian populations, with a focus on re-evaluation of the relationship between age at onset and functional outcomes in this population.

## Methods

### Inclusion criteria

The source population for this study was patients hospitalized at Peking University Institute of Mental Health in 2006. There was no bottom age limit for children’s admissions to this hospital. Patient records numbered 14,001 to 15,000 were consecutively assessed, and subjects were enrolled according to the following criteria:

1. Patient’s age did not reach 18 years old on day of admission.
2. Primary clinical diagnosis at discharge was “schizophrenia” (*n* = 113) or “schizophrenia likely” (*n* = 5). Diagnoses were all made according to ICD-10 criteria by 2 or more experienced psychiatrists.
3. Patients or family members have sufficient comprehension of Mandarin Chinese.

A total of 118 patients were identified according to the above criteria and included in our study.

### Data collection

Baseline information was extracted from inpatient medical records, including demographic data, patient history, family history, and information regarding initial hospitalization. These were obtained from family members during the clinical interview at admissions and were recorded by residents and approved by attending physicians. Relevant data were extracted for the present study by the primary researchers.

Follow up data was collected primarily via telephone interviews as most patients (66%) lived out-of-province. Points-of-contact were most commonly patients’ parents. Of the 118 subjects whose baseline data were collected, 48 were lost to follow up due to discontinued phone numbers, while 5 refused the interview. A total of 65 subjects were successful interviewed. The primary informant was a parent, the patient themselves, and another family member for 59, 3, and 3 subjects respectively.

Interviewees were asked to provide information to their best knowledge. In order to endure data quality, in cases where the interviewee did not live with the patient and was not willing to disclose patient contact information, facts such as employment and marriage status were collected, but subjective scales were not administered. For 5 subjects, a second family member was able to corroborate the information provided.

### Outcome variables

The primary outcome for this study was overall functioning at time of follow up. This was assessed using the World Health Organization Disability Assessment Schedule 2.0 (WHODAS 2.0), which has been validated in Chinese speaking populations [7]. The WHODAS comprehensively assesses daily functioning in multiple domains and has been shown to have good validity in people with schizophrenia [8]. The WHODAS is scored on a 0 to100-point scale with 0 being no functional impairment, and 100 being the highest possible level of impairment. A total of 57 subjects completed the WHODAS; incompletion was either due to time restraints on the interviewee’s side, or insufficient knowledge about patient’s current status.

Descriptive outcomes were also collected regarding education, employment, mortality, depression, suicide, physical health, subsequent treatment and changes in diagnosis, using the data collection form designed for this study. For physical health, primary informants were asked to report patient height and weight, as well as any comorbid diagnoses. They were specifically asked if hyperlipidemia, high blood sugar and hypertension were present, then asked to report any other physical comorbidities. Body Mass Index (BMI) was calculated using the formula: BMI = weight (kg)/[height (m)]^2^.

### Covariates

Two age-of-onset variables were used in the data analysis: onset of any symptoms was defined as the onset of any aberrations as described in the medical records, excluding symptoms of comorbid intellectual disabilities, attention-defict/hyperactively disorder and/or tic disorders. Onset of psychotic symptoms was recorded when possible for those whose records specify a time origin for the appearance of psychotic symptoms. In cases where there was acute onset of psychotic symptoms, the same time of onset was adopted for these two variables. There was inconsistency in the degree of detail for time of onset data in the medical records. Therefore, in cases where only the month or year of onset was available, dates were imputed by assuming the mid-point of the smallest time interval provided.

Premorbid personality traits were subjectively described by patient caregivers and recorded during the initial intake assessment. Therefore, the terminologies used were not standardized. In the analysis, similar personality traits were grouped into the following categories: extroverted, introverted, unsociable, suspicious, perfectionistic, obstinate, gentle and capricious. Because individuals were often described with multiple traits, each personality trait was classified as a binary variable.

Education was coded as a nominal categorical variable according to conventional classification in China, where elementary school = less than or equal to 6 years of education, middle school = 6–9 years of general education, mid-level vocational training = 9 years of general education plus up to 3 years of vocational education, high school = 9–12 years of general education, high level vocational training = 12 years of general education plus up to 3 years of vocational training, university = attended undergraduate education, post-graduate = attended post-graduate education. For regression analysis, education was dichotomized into two groups using 9 years of education as a cutoff. This cutoff was chosen both because it is the mandatory number of years of education for children in China, and also because the distribution for highest education was bimodal with a peak in the middle school group.

Antipsychotic medication dosage was transformed into olanzapine equivalent doses using the defined daily dose method [9].

### Data analysis

In the descriptive analysis, means and standard deviations were calculated for normally distributed variables. Two tailed Student’s t tests were used to compare outcomes between groups. Square root transformation of WHODAS score was used to achieve linearity in the regression analysis. Univariate regression models were used to explore unadjusted effects, and multivariate regression models were used in the risk factor assessment. Candidate covariates for the final model were chosen based on results from the univariate analysis and backwards stepwise regression. Likelihood ratio tests and Akaike Information Criteria were used to assist in determining the final model. Propensity scores were used to control for confounding in analyzing the relationships between age of onset and WHODAS score and BMI. We conducted a sensitivity analysis using two cut-offs: onset before age 13 and onset before age 14.7, the latter of which draws from Lin et al.’s findings that age 14.7 is a more optimal cut-off for differentiating function [10]. All inferences were made under a significance level of α = 0.05. Data analysis was conducted using Stata 15.0 (StataCorp).

## Results

### Baseline information

Baseline data were collected for 118 individuals. Our study population was 50% female. Mean age at onset of any symptoms was 13.7 (SD 2.3, range 7.1 to 17.7) years. For the 68 individuals with clear records of time of onset of psychotic symptoms, mean age at first presentation of psychotic symptoms was 14.4 (2.1, range 7.7 to 17.8) years. Participants mostly resided in urban cities (71%), while 29% lived in rural communities. Thirty percent of all participants had a family history of psychiatric disease. Psychiatric comorbidities were present in 11 subjects (9%), two of which had multiple comorbidities. At discharge, patients were on average using a 13.6 ± 6.9 mg olanzapine equivalent dosage of antipsychotic medication. Subjects were on average followed up for 10.4 ± 0.3 years, with a total of 686.8 person-years of follow up. At time of follow up, the average age of patients was 25.9 ± 2.1 years. The interviewed subjects and non-interviewed subjects did not differ significantly in baseline characteristics (Table 1).

**Table 1:** Baseline characteristics and comparison by follow up status

Variable	All Participants (*N* = 118)	Interviewed Group (*N* = 65)	Non-interviewed Group (*N* = 53)	*p*
Female, %	50%	49%	51%	0.85
Age at onset, y (SD, range)				
Any symptoms	13.7 (2.3, 7.1 to 17.7)	14.0 (2.2, 7.1 to 17.7)	13.4 (2.3, 7.1 to 16.8)	0.19
Psychiatric symptoms	14.4 (2.1, 7.7 to 17.8)	14.8 (2.2, 8.2 to 17.8)	14.1 (2.1, 7.7 to 16.9)	0.18
Number of VEOS, n (%)	39 (34%)	18 (29%)	21 (40%)	0.21
Urban residence, %	71%	67%	75%	0.36
Positive family history, %	30%	29%	30%	0.91
Presence of and comorbidity, %	9%	9%	9%	0.97
Comorbid ADHD, %	4%	5%	4%	0.82
Comorbid Tourette’s syndrome, %	3%	2%	4%	0.45
Comorbid other tic disorders, %	1%	2%	0%	0.37
Comorbid intellectual disabilities, %	3%	5%	2%	0.42
Presence of personality traits^a^, %				
Extroversion	22%	23%	21%	0.76
Introverted	70%	69%	72%	0.73
Unsociable	42%	38%	47%	0.35
Suspicious	28%	26%	30%	0.63
Perfectionistic	20%	17%	25%	0.31
Obstinate	12%	11%	13%	0.68
Gentle	7%	6%	8%	0.78
Capricious	8%	11%	4%	0.15
Antipsychotic dosage at discharge, olanzapine equivalent dose, mg (SD)	13.6 (6.9)	13.7 (6.2)	13.6 (7.8)	0.93

^a^Traits were non-exclusive

### Mortality, suicide and depression

Of the 65 patients about whom we succeeded to collect follow-up data, 3 were deceased at time of follow up. Cause of death was reported for two cases: suicide under depressive mood and suicide under the influence of psychotic symptoms. The third patient’s family did not wish to disclose cause of death. Of the surviving 62 patients, depression during the follow up period was reported in 12 (19%) individuals; 40 (65%) denied depression, while 10 (16%) could not provide information. Suicidal ideation and suicide attempts were endorsed by 24 (39%) and 5 (8%) subjects respectively.

### Diagnostic stability

Of the 55 subjects about whom we had information on subsequent diagnoses, 42 (76%) retained the original diagnosis while 13 were no longer diagnosed with schizophrenia. Six were re-diagnosed with bipolar disorder, 2 with depression, and one with schizoaffective disorder. One individual no longer carried a psychiatric diagnosis, one individual’s parents were told that their child had “mental retardation with psychotic symptoms”, and one individual retained their comorbid diagnoses of ADHD, tic disorder and obsessive-compulsive disorder but was no longer diagnosed with schizophrenia. Information was not available regarding the subsequent diagnosis for one individual.

### Treatment and medication

At time of follow up, 5 (8%) patients discontinued all formal psychiatric treatment, while 57 (92%) continued to use psychiatric services. Data on readmissions was available for 59 subjects. Twenty-five (40%) were never readmitted for psychiatric disorders, 17 (27%) were readmitted once, 10 (16%) were readmitted twice, and 7 (11%) were readmitted three or more times. Readmission was not associated with age of onset (OR = 0.95, *p* = 0.68), sex (OR = 0.62, *p* = 0.36), antipsychotic medication dosage at discharge (OR = 1.0, *p* = 0.56), or clozapine use at follow up (OR = 1.89, *p* = 0.34). Forty-eight (77%) of patients were regularly taking medication at follow up. The mean daily dose of antipsychotic medication at discharge and at follow up was 13.8 ± 6.2 mg and 11.9 ± 7.8 mg olanzapine equivalents respectively. There was no correlation between medication dosage at discharge and at time of follow up (Pearson’s r = 0.15).

**Fig. 1:**
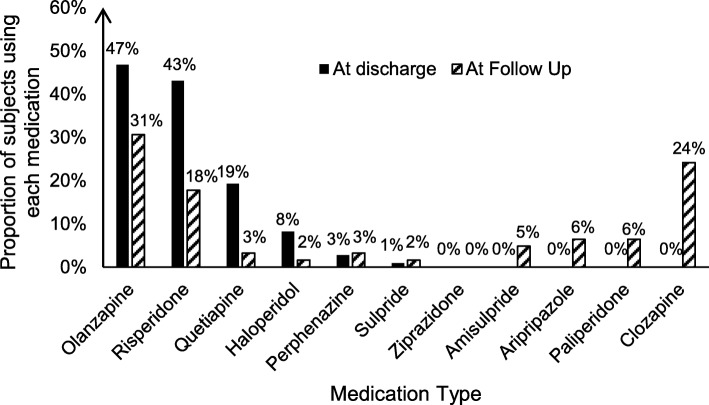
Change in antipsychotic medication prescription from baseline to follow up. On the horizontal axis is antipsychotic drug type, arranged in descending order from left to right by frequency of use at baseline. Data shown is the proportion of patients using each drug at baseline and follow up. Each patient could have been using multiple drugs.

Changes in the types of medication used was also evident (Fig. 1). At time of discharge, olanzapine (47%) and risperidone (43%) were by far the most common medications. At time of follow up, the proportion of patients on these two medications had decreased to 31 and 18% respectively, while the use of clozapine (24%) as well as newer second-generation antipsychotics such as paliperidone (6%) and aripiprazole (6%) have emerged.

### BMI and physical health

Data regarding patient height, weight and BMI are presented in Table 2. In male and female patients, 61 and 55% respectively exceeded the WHO standard for normal weight range (BMI ≥ 25 kg/m^2^), while 29 and 32% respectively reached the WHO threshold for obesity (BMI ≥30 kg/m^2^) [11]. In the univariate regression models, BMI at follow up was not significantly related to patient age of onset (B = 0.2, *p* = 0.18) or sex (B = − 1.4,*p* = 0.32), nor was it related to antipsychotic medication dosage either at discharge (B = 0.08, *p* = 0.45) or at follow up (B = 0.14, *p* = 0.09). BMI did not differ significantly in individuals using olanzapine compared to those who did not, whether at baseline (B = − 0.51, *p* = 0.72) or at follow up (B = 0.27, *p* = 0.85). Similar findings were found for risperidone at baseline (B = − 0.55, *p* = 0.70) and at follow up (B = 0.47, *p* = 0.80), quetiapine at baseline (B = 0.46, *p* = 0.79), and clozapine at follow up (B = 1.70, *p* = 0.27). Comorbid physical diseases were reported in 24 (39%) subjects. High blood lipid, fatty liver disease, elevated blood glucose, hypertension and hyperuricemia were reported in 8, 5, 4, 2 and 2 cases respectively.

**Table 2:** Height, Weight and BMI by Sex

	Male	Female
Height (x̄±SD)	1.75 ± 0.06 m	1.65 ± 0.06 m
Weight (x̄±SD)	86.0 ± 23.7 kg	71.4 ± 18.9 kg
BMI, n (%)		
Underweight (<18.49 kg/m^2^)	1 (3%)	2 (6%)
Normal Range (18.50–24.99 kg/m^2^)	11 (36%)	12 (39%)
Overweight (25.00–29.99 kg/m^2^)	10 (32%)	7 (23%)
Obese (> 30.00 kg/m^2^)	9 (29%)	10 (32%)

### Social outcomes

In our sample, 4 (6.5%) and 10 (33.9%) individuals respectively reached elementary and middle school education levels, while 5 (8.1%), 9 (14.5%) and 10 (16.1%) respectively have attended mid-level vocational training, high school, and high-level vocational training. A further 11 (17.7%) attended university, and 2 (3.2%) reached post-graduated level education. Highest education level was not available for one subject. Thirty-four (55%), 23 (37%) and 5 (8%) respectively were unemployed, currently employed and currently in school. Sixteen (26%) reported that the patient was economically self-sufficient. Thirteen (21%) patients had ever been married. Of note, the average age at follow up was 25.9 years.

### Patient functioning and associated factors

We used the WHO disability assessment scale to measure patient functioning at follow up. In our sample, WHODAS scores were highly right skewed. The median score is 15, with an IQR of 2 to 35, roughly corresponding to the 78th, 52nd, and 90th percentiles respectively on population norms [12].

Various baseline factors were analyzed for relationship with WHODAS score at follow up (Table 3). We found that extroverted personality (*p* = 0.01) and suspicious personality (*p* = 0.02) at baseline, as well as higher level of education (*p* = 0.001) were all independent factors related to improved functioning. Antipsychotic medication dosage at follow up was significantly associated with improved functioning, however the effect measure was not large enough to be of clinical significance (B = 0.01, 95% CI 0.00–0.03 in the final model, p = 0.01). Contrary to general impression, age of onset of any symptoms was not associated with function in either the univariate model (*p* = 0.24), the full model (*p* = 0.17) or the final risk factor model (*p* = 0.11). Notably, the effect measure of age in the full model was close to null (β = − 0.04).

**Table 3:** Factors related to WHODAS score at follow up

Risk factors	Univariate		Full model^a^		Final model	
	B (95% CI)	p	B (95% CI)	p	B (95% CI)	p
Sex	−0.30 (−3.70, 0.67)	0.43	0.02 (−0.93, 1.49)	0.82	0.00 (−1.13, 1.12)	0.99
Education	−7.44 (−15.62, −2.26)	< 0.001	0.02 (− 2.15, 3.08)	0.86	−4.19 (− 10.01, − 0.87)	0.001
Age at disease onset	− 0.02 (− 0.12, 0.01)	0.24	− 0.08 (− 0.49, 0.01)	0.17	−0.04 (− 0.18, 0.00)	0.11
Extroverted personality	−6.81 (− 16.79, − 1.25)	0.001	−4.09 (− 11.02, − 0.52)	0.004	−3.7 (− 10.56, − 0.35)	0.01
Suspicious personality	−1.30 (− 7.15, 0.15)	0.15	−2.77 (− 10.29, − 0.01)	0.04	−1.82 (− 6.11, − 0.05)	0.02
Antipsychotic medication dosage at follow up	0.01 (0.00, 0.04)	0.005	0.01 (0.00, 0.04)	0.01	0.01 (0.00, 0.03)	0.01
Clozapine usage at follow up	2.15 (0.00, 8.89)	0.06	1.57 (− 0.03, 7.12)			

^a^Model also adjusted for current medication status, psychiatric comorbidities, having any symptoms in the past 30 days, calm personality, number of hospitalizations, treatment effect at baseline, and whether a change was made to the original diagnosis

### Age of onset and outcome

To fully control for all potential confounders, we conducted a further analysis of the relationship between age and outcome using propensity scores. We found that after adjusting for sex, personality, education, rural or urban residence, having any psychiatric comorbidity, antipsychotic medication dosage at discharge, current use of any antipsychotic medication and having any symptoms in the past 30 days using propensity scores, neither age at onset of psychotic symptoms nor age at onset of any symptoms were associated with outcomes at follow up (Table 4). Changing the age cut-off to 14.7 did not alter this finding.

**Table 4:** Predictive value of different age cutoffs on outcome^a^

Outcome Variable	Age at Onset Cutoff Criteria		N in each bin		B	*p*
	Symptom Criteria	Age cutoff	<cutoff	≥cutoff		
WHODAS	Any symptoms	14.7	27	21	−1.1	0.18
	Any symptoms	13.0	11	34	−0.9	0.37
	Psychotic symptoms^b^	14.7	10	11	−0.3	0.67
BMI	Any symptoms	14.7	29	18	0.7	0.68
	Any symptoms	13.0	13	33	1.5	0.42
	Psychotic symptoms	14.7	11	10	−2.2	0.30

^a^Controlled for sex, extroverted personality, suspicious personality, currently on medication, education, any comorbidity, any symptoms in the past 30 days, rural residence, family disease history, and antipsychotic dosage at discharge using propensity scores

^b^Only one cutoff (age 14.7) is used for the onset of psychotic symptom models because the models using age 13.0 did not converge

## Discussion

To date, there have been few longitudinal studies regarding the long term (> 5 years) outcome of EOS, especially in low and middle-income countries. The current study describes in detail multiple outcomes of EOS at 10 years after discharge from a tertiary psychiatric hospital in China, with a focus on the relationship between age at onset and functional outcome.

### Mortality and suicide

At 10 years post discharge, mortality in our study population was 4.6%. Cause of death was suicide for two cases, and unconfirmed for the remaining case. The mortality rate was 4.3 per 1000 person-years. Previous studies in Chinese populations have reported comparable suicide rates of 6.8 per 1000 person-years [13] and 4.77 per 1000 person-years [14]. However, methodological differences between studies limit comparability. Because our study population is relatively young (on average 25.9 years old), there is potential for future cases of suicides and accidental deaths as time moves on. Whether EOS patients have increased excess mortality compared to adult onset cases warrants further attention.

### Diagnostic stability

The diagnosis of EOS has been found to be relatively stable over time. In an early study by Hollis, the positive predictive value of an EOS diagnosis was 80% at 11 years post disease onset [15]. Two small studies which included 18 and 9 subjects respectively both found a 100% positive predictive value of EOS diagnosis, the first at 10 years post hospital admission, the second at 28 years after baseline [16, 17]. A recent study in Yunnan province of China found that 92% of patients retained their original diagnosis of schizophrenia after an average 1.5 years of follow up [4]. In our study, 76% of individuals were still diagnosed with schizophrenia at follow up, similar to the number reported by Hollis and lower than numbers of Jarbin, Helgel and Kang. Jarbin and Helgel and had smaller sample sizes and excluded VEOS cases, while Kang et al. followed up subjects for only a short period of time, which may explain the higher diagnostic stability.

Notably, 6 of the 13 individuals whose diagnosis changed were later diagnosed with bipolar disorder in our study. This result is similar to previous studies. Previous follow up studies found an increase from 12.2% at baseline to 24.5% at follow up in the diagnosis of affective disorder in children with psychosis [18]. It has also been previously reported that differential diagnosis between pediatric bipolar disorder and schizophrenia is difficult due to overlapping phenomenology [19, 20]. Another study found that in children with first episode psychosis, 23, 41 and 19% respectively have depressive, mixed and manic symptoms [21]. These findings highlight the limitations of clinical assessments based on one time point in children with psychosis and the need for continued follow up to improve the accuracy of diagnoses.

### Trends in medication

There is increased variety in the types of second-generation antipsychotic medication used in the follow up sample of our study. Notably, rates of clozapine use reached 24%, higher than the numbers reported in a registry based study from Denmark which found that clozapine was prescribed to 17.6% of all EOS patients [22]. This discrepancy is likely due to variations in prescriptions habits in different institutions across the world, as well as cost of treatment. In China, high rates of clozapine use are especially noticeable in areas with lower economic development. In 2002, 28% of all patients were prescribed clozapine in a hospital in Beijing, while this number was 62% in a less developed district [23]. In more recent years, rates of clozapine use across the country have decreased, but were still as high as 26.4% in 2012 [24]. In our study population, 34% did not permanently reside in Beijing, and 29% were from rural communities. Therefore, they would likely be receiving outpatient treatment in areas with traditionally high rates of clozapine prescription. Furthermore, insurance rates for children are lower than adults in our country. One study showed that only 64% of children were insured [25]. Even when insurance is available, in our clinical experience, many parents choose to not use it because they do not wish psychiatric diagnoses to appear on their child’s records. Therefore, cost is an important factor that may prohibit the long-term use of newer, more expensive antipsychotics and increase the rate of clozapine prescription in our study.

### Obesity and physical health

High BMI was a prominent issue in our study population. At follow up, 61 and 55% of male and female patients respectively exceeded the WHO standard for normal weight range, while 29 and 32% respectively were obese. In comparison, in the general population in China, the proportion of overweight adults in 2013 was only 28.3% for men and 27.4% for women, while obesity was only present in 3.8 and 5.0% of men and women respectively [26]. Multiple short-term studies have shown that antipsychotic related weight gain occurs within the first year of medication in EOS populations [27, 28] and can reach as high as 7.3 kg in the first 8 weeks of olanzapine therapy [29]. However, obesity related outcomes have not been reported in previous long term follow up studies [2, 3, 6, 30]. Weight gain and obesity contribute to metabolic syndrome and cardiovascular morbidity, which is currently believed to be the major contributor to excess mortality in schizophrenia [31]. For EOS populations, the harmful effects of weight gain are likely greater than for adult onset patients, as they become exposed to this risk factor at an earlier age. Though our study population was relatively young at follow up, metabolic abnormalities including high blood lipid, fatty liver disease, elevated blood glucose and hypertension were already prevalent. These results highlight the need for more studies regarding the extent and potential interventions of weight gain in EOS populations.

### Functional outcomes and related risk factors

Similar to previous studies [2, 3, 5], we found significant heterogeneity in the distribution of functional outcome at follow up. In previous reviews, the overall outcome of early onset schizophrenia was concluded to be poor [2, 30]. However, early studies generally utilized unstandardized clinical criteria or simple global measures such as the Global Assessment of Functioning (GAF) and the Children’s Global Assessment Scale (CGAS) for assessment of patient functioning. Using the comprehensive WHO Disability Assessment Schedule, our results were somewhat more optimistic: a quarter of the individuals in our study scored above the 52% percentile of overall functioning compared to population norms.

In the risk factor analysis, compared to those with lower education levels, those who continued education beyond middle school had significantly better functioning. Patients who were described as outgoing, extroverted or optimistic were more likely to have better long-term outcomes. Surprisingly, patients who were described as suspicious also had better outcomes. We hypothesize that this may be because delusions, which often evolve from suspicions, are positive symptoms which are generally responsive to treatment. Our finding that age at onset in EOS was not associated with long term outcome differs from conventional beliefs. In our risk factor analysis, the effect measure of age on WHODAS score is sufficiently insignificant (− 0.04 in the final model) that it is unlikely this result was due to low power. Using propensity score analysis to control for confounding, we found that neither of the two age of onset variables used were associated with long term outcome. A sensitivity analysis using an alternate age cut-off showed similar results.

Because VEOS is extremely rare with an estimated prevalence of less than 0.04% [32], there are few studies that directly compare its outcome with that of EOS. A recent meta-analysis found that age at onset was not a consistent risk factor for poor outcome in multivariate models, especially when only adolescent subjects were included [33]. Our robust analysis shows that there is no difference in long term functional outcomes between EOS and VEOS, using either the classic age cut-off or the 14.7 year old cutoff proposed by Lin et al. [10], nor when analyzed as a continuous variable. To our knowledge, this is the first study directly comparing long term prognosis between EOS and VEOS using standardized measurements of functioning. Our findings call to question the long-standing assumption that earlier age at onset is associated with poor outcomes in EOS.

### Limitations

An important limitation of this study is its retrospective design. Baseline data were acquired through chart review and were not recorded according to research standards, which may result in vague terminologies and insufficient detail. In addition, interviewees were asked to retrospectively recall treatment history, which may have resulted in data incompleteness and inaccuracy.

There was relatively high loss to follow up rate in our study, mostly due to discontinued phone numbers, a non-differential cause of loss of follow up; only 5 families refused to participate in the study. Furthermore, baseline characteristics of those lost to follow up did not differ with those successfully interviewed. This reduces the likelihood that the high attrition rate has substantially biased the outcome.

Additionally, this study relied heavily on proxy report, which is influenced by various subjective factors and thus may result in inaccurate information, especially in estimating patient functioning. As Chinese families remain extremely protective of their children even into their early adulthoods, most parents refused to allow us to directly contact patients. The interview method also restricted our ability to obtain subjective measures regarding physical comorbidities. This may result in underestimation of metabolic diseases because many subjects did not receive regular medical evaluations.

Another limitation of our study is its sampling method. The relatively small sample size limited our ability to perform more in-depth statistical analyses and may have resulted in insufficient power in hypothesis testing. Also, data from a single center study lacks generalizability to other patient populations. The hospital where this study was conducted was a tertiary hospital in a highly developed part of China. Our patient population is more likely to be of high socioeconomic backgrounds and to have failed previous treatments at other hospitals. Furthermore, using inpatient records precluded patients whose symptoms were not severe enough to warrant hospitalization. Because of this, our study population does not represent the whole spectrum of disease severity in EOS, especially those with less functional impairment.

Although our study yielded numerous findings, because of the limitations mentioned above, our results require further confirmation. Larger prospective cohort studies are indicated to improve our understanding of the outcomes of EOS and its predictors, especially age of disease onset.

### Implications

Correctly identifying independent risk factors related to long term disease outcome is crucial for the planning of treatment services and resource allocation, as well as for the expectation management of each afflicted family. Our study found that the long-term outcomes of EOS vary widely and is more optimistic than previously assumed. We also found that extroverted personality, suspicious personality, and higher level of education were associated with better functioning at follow up. Furthermore, our analysis showed that age at onset is not a significant predictor of functional outcome in EOS populations. The findings from this preliminary study increase our understanding of the functional outcomes of EOS and its risk factors and can hopefully provide reference for future prospective studies about EOS prognosis.

## Data Availability

De-identified data used in this study can be made available upon request. Inquiries can be sent to the corresponding authors.

## Abbreviations

BMI: Body Mass Index
CGAS: Children’s Global Assessment Scale
EOS: Early Onset Schizophrenia
GAF: Global Assessment of Functioning
VEOS: Very Early Onset Schizophrenia
WHODAS: World Health Organization Disability Assessment Schedule

## References

[1] Remschmidt H, Theisen F (2012). Early-onset schizophrenia. Neuropsychobiology.

[2] Fleischhaker C, Schulz E, Tepper K, Martin M, Hennighausen K, Remschmidt H (2005). Long-term course of adolescent schizophrenia. Schizophr Bull.

[3] Remschmidt H, Martin M, Fleischhaker C, Theisen FM, Hennighausen K, Gutenbrunner C, Schulz E (2007). Forty-two-years later: the outcome of childhood-onset schizophrenia. J Neural Transm (Vienna).

[4] Kang C, Zhou H, Yang J, Yang R, Sun N, Wang S, Yang C, Han D, Srihari VH (2018). Course, outcome and diagnosis stability of early-onset schizophrenia in Yunnan Province, China-a three years follow-up study. Psychiatry Res.

[5] Ropcke B, Eggers C (2005). Early-onset schizophrenia: a 15-year follow-up. Eur Child Adolesc Psychiatry.

[6] Reichert A, Kreiker S, Mehler-Wex C, Warnke A (2008). The psychopathological and psychosocial outcome of early-onset schizophrenia: preliminary data of a 13-year follow-up. Child Adolesc Psychiatry Ment Health.

[7] Chiu TY, Yen CF, Chou CH, Lin JD, Hwang AW, Liao HF, Chi WC (2014). Development of traditional Chinese version of World Health Organization disability assessment schedule 2.0 36--item (WHODAS 2.0) in Taiwan: validity and reliability analyses. Res Dev Disabil.

[8] Sjonnesen K, Bulloch AG, Williams J, Lavorato D, Patten SB (2016). Characterization of disability in Canadians with mental disorders using an abbreviated version of a DSM-5 emerging measure: the 12-item WHO disability assessment schedule (WHODAS) 2.0. Can J Psychiatr.

[9] Leucht S, Samara M, Heres S, Davis JM (2016). Dose equivalents for antipsychotic drugs: the DDD method. Schizophr Bull.

[10] Lin A, Wardenaar KJ, Pontillo M, De Crescenzo F, Mazzone L, Vicari S, Wood SJ, Beavan A, Armando M (2016). Is it still correct to differentiate between early and very early onset psychosis?. Schizophr Res.

[11] WHO (2015). Global Reference List of 100 Core Health Indicators, 2015.

[12] Ustun TB, Kostanjsek N, Chatterji S, Rehm J. Measuring health and disability : manual for WHO disability assessment schedule (WHODAS 2.0). Geneva: World Health Organization; 2010.

[13] Phillips MR, Yang G, Li S, Li Y (2004). Suicide and the unique prevalence pattern of schizophrenia in mainland China: a retrospective observational study. Lancet.

[14] Ran MS, Chen EY, Conwell Y, Chan CL, Yip PS, Xiang MZ, Caine ED (2007). Mortality in people with schizophrenia in rural China: 10-year cohort study. Br J Psychiatry.

[15] Hollis C (2000). Adult outcomes of child- and adolescent-onset schizophrenia: diagnostic stability and predictive validity. Am J Psychiatry.

[16] Helgeland MI, Torgersen S (2005). Stability and prediction of schizophrenia from adolescence to adulthood. Eur Child Adolesc Psychiatry.

[17] Jarbin H, von Knorring AL (2003). Diagnostic stability in adolescent onset psychotic disorders. Eur Child Adolesc Psychiatry.

[18] Stentebjerg-Olesen M, Pagsberg AK, Fink-Jensen A, Correll CU, Jeppesen P (2016). Clinical characteristics and predictors of outcome of schizophrenia-Spectrum psychosis in children and adolescents: a systematic review. J Child Adolesc Psychopharmacol.

[19] Pavuluri MN, Herbener ES, Sweeney JA (2004). Psychotic symptoms in pediatric bipolar disorder. J Affect Disord.

[20] Singh MK, Ketter T, Chang KD (2014). Distinguishing bipolar disorder from other psychiatric disorders in children. Curr Psychiatry Rep.

[21] Sanchez-Gistau V, Baeza I, Arango C, Gonzalez-Pinto A, de la Serna E, Parellada M, Graell M, Paya B, Llorente C, Castro-Fornieles J (2015). The affective dimension of early-onset psychosis and its relationship with suicide. J Child Psychol Psychiatry.

[22] Schneider C, Papachristou E, Wimberley T, Gasse C, Dima D, MacCabe JH, Mortensen PB, Frangou S (2015). Clozapine use in childhood and adolescent schizophrenia: a nationwide population-based study. Eur Neuropsychopharmacol.

[23] Tang YI, Mao PX, Jiang F, Chen Q, Wang CY, Cail ZJ, Mitchell PB (2008). Clozapine in China. Pharmacopsychiatry.

[24] Li Q, Xiang YT, Su YA, Shu L, Yu X, Correll CU, Ungvari GS, Chiu HFK, Ma C, Wang GH, Bai PS, Li T, Sun LZ, Shi JG, Chen XS, Mei QY, Li KQ, Si TM, Kane JM (2015). Clozapine in schizophrenia and its association with treatment satisfaction and quality of life: findings of the three national surveys on use of psychotropic medications in China (2002-2012). Schizophr Res.

[25] Xiong JY, Hipgrave D, Myklebust K, Guo SF, Scherpbier RW, Tong XT, Yao L, Moran AE (2013). Child health security in China: a survey of child health insurance coverage in diverse areas of the country. Soc Sci Med.

[26] Ng M, Fleming T, Robinson M, Thomson B, Graetz N, Margono C, Mullany EC, Biryukov S, Abbafati C, Abera SF, Abraham JP, Abu-Rmeileh NM, Achoki T, AlBuhairan FS, Alemu ZA, Alfonso R, Ali MK, Ali R, Guzman NA, Ammar W, Anwari P, Banerjee A, Barquera S, Basu S, Bennett DA, Bhutta Z, Blore J, Cabral N, Nonato IC, Chang JC, Chowdhury R, Courville KJ, Criqui MH, Cundiff DK, Dabhadkar KC, Dandona L, Davis A, Dayama A, Dharmaratne SD, Ding EL, Durrani AM, Esteghamati A, Farzadfar F, Fay DF, Feigin VL, Flaxman A, Forouzanfar MH, Goto A, Green MA, Gupta R, Hafezi-Nejad N, Hankey GJ, Harewood HC, Havmoeller R, Hay S, Hernandez L, Husseini A, Idrisov BT, Ikeda N, Islami F, Jahangir E, Jassal SK, Jee SH, Jeffreys M, Jonas JB, Kabagambe EK, Khalifa SE, Kengne AP, Khader YS, Khang YH, Kim D, Kimokoti RW, Kinge JM, Kokubo Y, Kosen S, Kwan G, Lai T, Leinsalu M, Li Y, Liang X, Liu S, Logroscino G, Lotufo PA, Lu Y, Ma J, Mainoo NK, Mensah GA, Merriman TR, Mokdad AH, Moschandreas J, Naghavi M, Naheed A, Nand D, Narayan KM, Nelson EL, Neuhouser ML, Nisar MI, Ohkubo T, Oti SO, Pedroza A, Prabhakaran D, Roy N, Sampson U, Seo H, Sepanlou SG, Shibuya K, Shiri R, Shiue I, Singh GM, Singh JA, Skirbekk V, Stapelberg NJ, Sturua L, Sykes BL, Tobias M, Tran BX, Trasande L, Toyoshima H, van de Vijver S, Vasankari TJ, Veerman JL, Velasquez-Melendez G, Vlassov VV, Vollset SE, Vos T, Wang C, Wang X, Weiderpass E, Werdecker A, Wright JL, Yang YC, Yatsuya H, Yoon J, Yoon SJ, Zhao Y, Zhou M, Zhu S, Lopez AD, Murray CJ, Gakidou E (2014). Global, regional, and national prevalence of overweight and obesity in children and adults during 1980-2013: a systematic analysis for the global burden of disease study 2013. Lancet.

[27] O'Donoghue B, Schafer MR, Becker J, Papageorgiou K, Amminger GP (2014). Metabolic changes in first-episode early-onset schizophrenia with second-generation antipsychotics. Early Interv Psychiatry.

[28] Kumra S, Oberstar JV, Sikich L, Findling RL, McClellan JM, Vinogradov S, Charles Schulz S (2008). Efficacy and tolerability of second-generation antipsychotics in children and adolescents with schizophrenia. Schizophr Bull.

[29] Taylor JH, Jakubovski E, Gabriel D, Bloch MH (2018). Predictors and moderators of antipsychotic-related weight gain in the treatment of early-onset schizophrenia Spectrum disorders study. J Child Adolesc Psychopharmacol.

[30] Clemmensen L, Vernal DL, Steinhausen HC (2012). A systematic review of the long-term outcome of early onset schizophrenia. BMC Psychiatry.

[31] Olfson M, Gerhard T, Huang C, Crystal S, Stroup TS (2015). Premature mortality among adults with schizophrenia in the United States. JAMA Psychiatry.

[32] Driver DI, Gogtay N, Rapoport JL (2013). Childhood onset schizophrenia and early onset schizophrenia spectrum disorders. Child Adolesc Psychiatr Clin N Am.

[33] Diaz-Caneja CM, Pina-Camacho L, Rodriguez-Quiroga A, Fraguas D, Parellada M, Arango C (2015). Predictors of outcome in early-onset psychosis: a systematic review. NPJ Schizophr.

